# Effectiveness and Safety of Atazanavir Use for the Treatment of Children and Adolescents Living With HIV: A Systematic Review

**DOI:** 10.3389/fped.2022.913105

**Published:** 2022-05-23

**Authors:** Laura Saint-Lary, Marc Harris Dassi Tchoupa Revegue, Julie Jesson, Françoise Renaud, Martina Penazzato, Claire L. Townsend, John O'Rourke, Valériane Leroy, WHO HIV Hepatitis and STIs (HHS) Department

**Affiliations:** ^1^CERPOP, Inserm, Université de Toulouse, Université Paul Sabatier III, Toulouse, France; ^2^Global HIV, Hepatitis and STIs Programme, World Health Organization, Geneva, Switzerland; ^3^World Health Organization, Geneva, Switzerland

**Keywords:** adolescents, atazanavir, children, HIV, effectiveness, safety, systematic review

## Abstract

**Background:**

Atazanavir/ritonavir is recommended as a preferred second-line antiretroviral regimen in children older than 3 months, alternatively to lopinavir/ritonavir. We performed a systematic review to assess safety and effectiveness of atazanavir use in children and adolescents.

**Methods:**

We searched observational studies and clinical trials on Web of Science, Embase and Cochrane CENTRAL database between 2009/01/01 and 2020/10/01; as well as grey literature. We extracted safety (adverse events, grade 3 or 4 adverse events, treatment discontinuation) and effectiveness (CD4 cell counts and HIV viral load) outcomes. We estimated weighted summary pooled incidence with corresponding 95% confidence intervals.

**Results:**

Out of the 1,085 records screened, we included five studies (one comparative cohort, three single phase 2-3 trial arms, one retrospective cohort) reporting 975 children and adolescents, of whom 56% (544) received atazanavir. Three studies reported all-cause treatment discontinuation rates, yielding a pooled incidence of 19% [15–22] at 12 months. The comparative cohort compared atazanavir to darunavir, with few grade 3–4 adverse events, except transient hyperbilirubinemia, occurring in half (92/188) of the atazanavir patients. No death occurred (two studies reporting). Four studies described increased CD4 cell counts and decreased HIV viral load at 6 or 12 months.

**Conclusion:**

Few safety and effectiveness data were available for children and adolescents exposed to atazanavir. Transient grade 3–4 hyperbilirubinemia was the main adverse outcome reported. Immune and viral responses were descriptive. The use of atazanavir/ritonavir in children and adolescents needs further investigation, but remains a suitable option for a preferred second-line antiretroviral regimen.

**PROSPERO number:**

CRD42022309230

## Introduction

In 2020, 1.7 million children and adolescents aged 0–14 years were infected by the Human Immunodeficiency Virus (HIV) worldwide, of whom 1.1 million lived in Sub-Saharan Africa (1). AIDS-related deaths were reported in 99,000 children and adolescents, and 1,50,000 new HIV infections occurred in this population (1). The introduction and increased availability of antiretroviral drug therapy since the 2000s dramatically reduced HIV-related morbidity and mortality among children and adolescents (2, 3). However, in 2020, only 54% of children and adolescents received antiretroviral drugs (4). Antiretroviral therapy initiation is currently recommended in all children and adolescents living with HIV, regardless of World Health Organization (WHO) clinical stage or CD4 cell count (3). Since 2019, the WHO recommends a dolutegravir-based regimen in children and adolescents (aged more than 4 weeks and weighing more than 3 km), or a raltegravir-based regimen in neonates (aged more than 4 weeks) living with HIV, as the preferred first-line antiretroviral regimens, due to improved efficacy and safety compared to previous antiretroviral regimens (5). Atazanavir/ritonavir is recommended as a preferred second-line antiretroviral regimen in children and adolescents older than 3 months as an alternative option to lopinavir/ritonavir (5). Atazanavir/ritonavir is an attractive second-line option for two reasons: thanks to once daily dosing, capsule and oral powder formulations of atazanavir/ritonavir are better tolerated than lopinavir by children. In addition, it had less serum lipids effects compared to other protease inhibitor drugs (6–8). Some studies conducted among adults have reported increased levels of bilirubin after exposure to atazanavir, as well as nephrolithiasis (9–12). To our knowledge, no systematic review has been conducted to summarize the safety and effectiveness data of atazanavir use in the treatment of neonates, children and adolescents living with HIV.

To inform the update of antiretroviral treatment guidelines by the WHO in 2021, we performed a systematic review to assess safety and effectiveness of atazanavir used in first, second, or subsequent-lines of treatment of children and adolescents living with HIV.

## Methods

### Search Strategy

A systematic review was conducted according to the Center for Reviews and Dissemination (CRD) guidance (13), and the results were reported according to the Preferred Reporting Items for Systematic Review and Meta-Analysis (PRISMA) guidelines (14). The protocol is registered with PROSPERO, CRD42022309230. The search strategies are presented in the additional file 1. Searches were conducted on Web of Science, Embase and Cochrane Central Register of Controlled Trials (CENTRAL) databases for published studies. For the gray literature, we searched clinical trial registries updated in the past 2 years (ClinicalTrials.gov; WHO International Clinical Trials Registry Platform; EudraCT), the references from the most recent international guidelines on HIV treatment [WHO antiretroviral guidelines (6), US National Institutes of Health Guidelines for the Use of Antiretroviral Agents in Pediatric HIV Infection 2020 (15), Penta 2015, 2016, and 2019 guidelines (16–18)] and in conference abstract books (International AIDS Society Conference 2019 and 2020, Conference on Retroviruses and Opportunistic Infections 2019 and 2020, International Workshop on HIV Pediatrics 2018 and 2019, International Conference on AIDS and STIs in Africa 2019).

### Selection Criteria

We searched experimental, randomized or not, and observational studies, comparative or not published between 2009/01/01 and 2020/10/01, which documented atazanavir exposure in children and adolescents, aged 0 to 19 years, treatment-naïve or experienced, living with HIV. Atazanavir could have been used with any other antiretroviral drug recommended for HIV treatment of children and adolescents. Children exposed to antiretroviral drugs through breastfeeding were excluded.

Titles and abstracts were independently screened by two reviewers (LSL and MHD), as well as full texts of identified abstracts and discrepancies were resolved by discussing with a third reviewer (VL).

### Outcome Definition

In this study, we evaluated safety and effectiveness of atazanavir use in children and adolescents living with HIV. Safety outcomes included all treatment discontinuation, adverse events, or grade 3 or 4 adverse events. Adverse events and grade 3 or 4 adverse events were defined according to the National Institutes of Health definitions (19). An adverse event was defined as any unexpected or unfavorable sign in patients, including any abnormal laboratory finding, symptom or disease temporally associated with the use of a medical treatment, and in case of antiretroviral exposure, leading to discontinuation or interruption of treatment. A grade 3 or 4 adverse event was considered as a severe adverse event, including potentially life-threatening event, significant disability or incapacity, requirement or prolongation of hospitalization, and death (19). Effectiveness was assessed 6 months or more after antiretroviral initiation or switch, and based on the CD4 cell count increase and HIV viral load suppression using a threshold of 400 copies/mL or 50 copies/mL.

### Quality Assessment and Data Extraction

Reviewers (LSL, MHD, JJ and VL) were not blinded to the names of the authors, institutions, journal of publication or results of the studies. We used the Cochrane Risk of Bias tool version 2 (20) for randomized clinical trials and the tool developed by the Clinical Advances through Research and Information Translation (CLARITY) group for observational studies (21).

Data extracted were related to study characteristics (first author, publication year, study design, source of data, country, median duration of follow-up, number of included patients, inclusion and exclusion criteria), patient characteristics at baseline (age, sex, perinatally acquired HIV, treatment experience, tuberculosis coinfection, WHO stage, CD4 cell count or percentage, and HIV viral load), antiretroviral treatment details (treatment, dose, frequency, formulation and intervention/comparator group), safety (treatment discontinuation, adverse outcomes, grade 3 or 4 adverse outcomes) and effectiveness (CD4 cell count and viral load) data.

### Statistical Analysis

Study outcomes were described within a narrative synthesis. If more than two studies documented the same outcome and the incidences were statistically homogeneous using the Q chi-squared and the I^2^ statistics (*p* > 10%), a pooled-analysis was conducted to estimate a weighted summary pooled incidence and corresponding 95% confidence intervals (95%CI), using a random-effect model. Statistical analyses were performed using STATA® 16.1 software.

## Results

**Figure 1:**
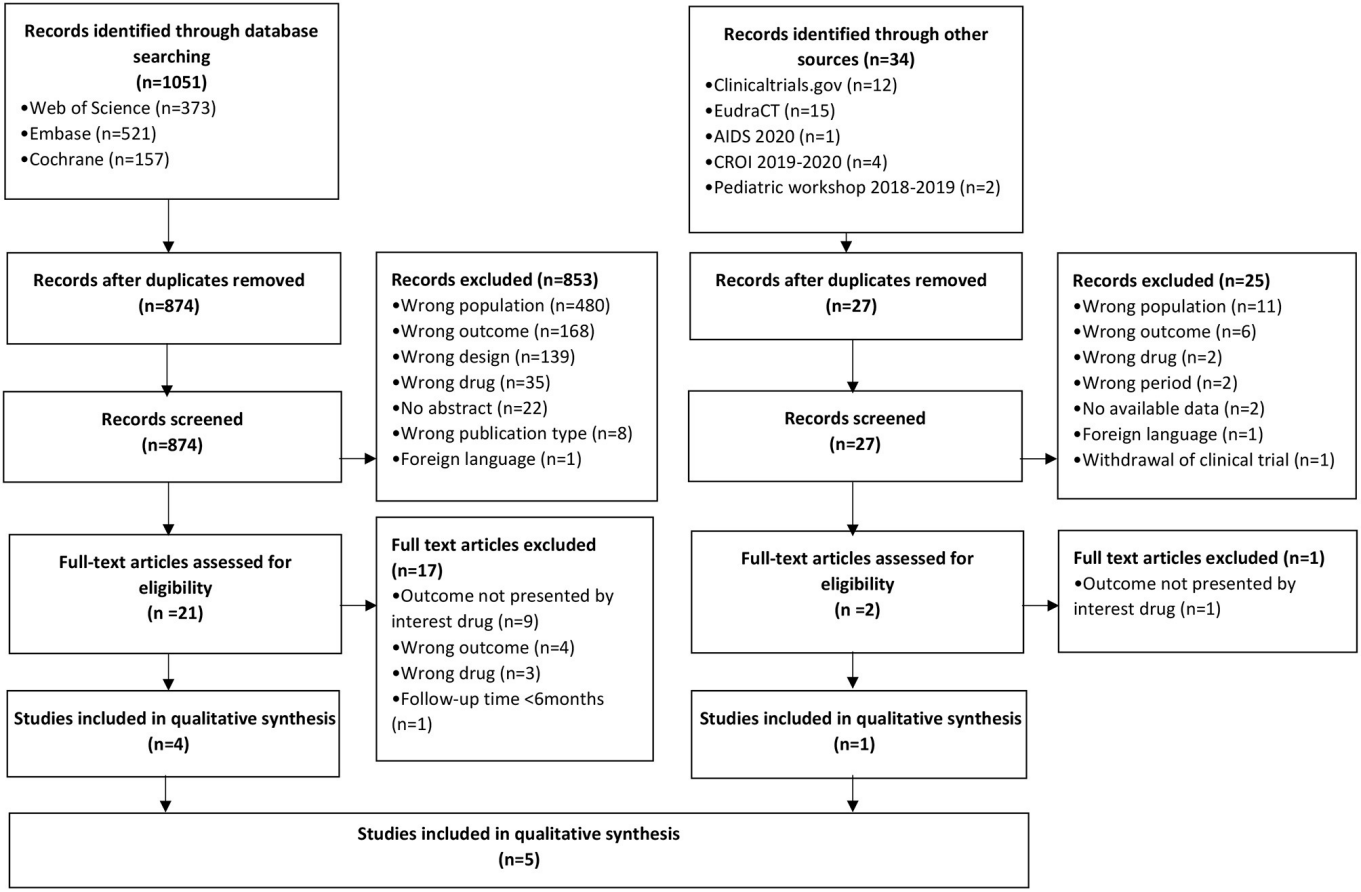
PRISMA flow diagram of study selection on atazanavir toxicity in children and adolescents (2009/01/01- 2020/10/01). CROI, Conference on Retroviruses and Opportunistic Infection.

Among the 1.085 records identified, 901 titles and abstracts were screened after duplicates removed. Overall, 23 full papers were assessed for eligibility, of which five were included in the narrative synthesis (Figure 1) (22–26).

Those five studies contributed data for 975 children and adolescents, of whom 544 (56%) received atazanavir. Three were observational cohort studies (22, 23, 25) (two retrospective and one prospective comparative, based on a phase 3b clinical trial) and two were prospective non-randomized clinical trials (24, 26). The single comparative cohort study (23) compared atazanavir-based with darunavir-based regimens, and was conducted in Europe and Thailand between 2011 and 2014. Two studies reported data in children [median age of 2.4 (22) and 3.4 years (24)], while the three others studies were conducted in adolescents (median age of 9.5 to 16.0 years) (23, 25, 26). Three studies included a mixture of antiretroviral-naïve and antiretroviral experienced children (9.0% to 60.7% were treatment naïve at inclusion) (22–24). Several antiretroviral drugs were used in addition to atazanavir: one study assessed the effect of lamivudine, fosamprenavir, atazanavir/ritonavir (25); the other studies assessed the effects of atazanavir associated with two nucleoside reverse transcriptase inhibitors (NRTI). Among NRTIs, tenofovir, abacavir, lamivudine, zidovudine, didanosine and stavudine were used. Didanosine was used in two studies, in 3/10 (30%) (26) and 2/56 (3.6%) (22) patients, and stavudine in 3/10 (30%) (26) and 4/56 (7.1%) (22) cases. The study periods ranged from 2009 to 2014. Regarding the follow-up duration, three studies were conducted over a 12-month period (48-weeks) (22–24), one over a 6-month period (26) and one over a 42-month period (25). The studies were implemented in various settings: Europe (*n* = 3), Sub-Saharan Africa (*n* = 3), Thailand (*n* = 2), Latin America (*n* = 2), United States of America (*n* = 2), and Russia (*n* = 1). Study characteristics are summarized in Table 1.

**Table 1:** Characteristics of the five studies included and their study population in the atazanavir systematic review (2009/01/01–2020/10/01).

**Study**	**Setting**	**Period**	**Study design**	**Length of follow–up**	**Number of patients**		**Median age [IQR] and sex**	**ART regimen (comparison group)**	**ART experienced or naïve at inclusion**
					**Total**	**on ATV**			
Piatt et al. (26)	Uganda	2009–2013	Prospective, non-comparative, open–label clinical trial	6 months	10	10	9.5 years [6.0–18.0] Male *n* = 80% Female *n* = 20%	ATV/r (All patients were on LPV/r at baseline and switched to ATV/r due to high lipid level)	ART experienced
Cotton et al. (24)	Argentina, Brazil, Chile, Mexico, Poland, Romania, Russia, South Africa, Spain and the US	2011–2014	Prospective, international, non-comparative, single arm phase 3b clinical trial	12 months	99	99	3.4 years [0.25–10.0] Male *n* = 49% Female *n* = 51%	ATV/r (No comparator)	ART–experienced (62.6%) and naïve (37.4%)
Rusconi et al. (25)	Italy	2011	Retrospective non-comparative cohort	42 months	7	7	16.0 years [10.8–18.9] Male *n* = 71% Female *n* = 29%	ATV–FAPV/r (No comparator)	ART–experienced
Strehlau et al. (22)	Brazil, Chile, Mexico, Peru, South Africa and Thailand	2010–2014	Prospective non-comparative cohort based on a phase 3b clinical trial	12 months	56	56	2.4 years [0.25–5.4] Male *n* = 50% Female *n* = 50%	ATV/r (No comparator)	ART–experienced (39.3%) and naïve (60.7%)
Bailey et al. (23)	Europe and Thailand	2011–2014	Retrospective–comparative cohort	12 months	803	372	13.5 years [11.4–15.2] Male *n* = 44% Female *n* = 56%	ATV (DRV)	ART–experienced (91.0%) and naïve (9.0%)

*ART, Antiretroviral drugs; ATV, Atazanavir; DRV, Darunavir; FAPV/r, Fosamprenavir/ritonavir; HIV, Human Immunodeficiency Virus; IQR, Interquartile Range; LPV/r, Lopinavir/ritonavir*.

### Safety of Atazanavir Use in Children and Adolescents Living With HIV

The results of atazanavir safety and effectiveness are presented in Table 2.

**Table 2:** Description and summary results for each study included in the systematic review (2009/01/01–2020/10/01).

**Study**	**Safety**						**Efficacy**	
	**Treatment discontinuation**		**Grade 3 or 4 adverse drug reactions**		Other adverse events^*^	Mortality^*^	CD4 count^*^	Viral load^*^
	**(reasons and time)**							
	**ATV**	**ARV**	**ATV**	**ARV**				
Piatt et al. (26)	NA	NA	Grade 3/4 adverse drug reactions: 10% [0–29] Grade 3/4 Elevated ALT: 0% Grade 3 bilirubin: 1/10	NA	Triglycerides level: from a mean of 288.5 at baseline to 193.3 at 6 months (NS) Cholesterol level from a mean of 214.3 at baseline to 178.5 at 12 weeks (*p* = 0,006)	NA	Mean absolute number (sd) of CD4 count at 6 months: 1120 (413.2)	No HIV viral failure at 6 months (≥200 copies/mL) Mean HIV VL at 6 months (log copies/mL): 2.6 (8.2)
Cotton et al. (24)	All causes 18% [11–26] Related reasons: • Adverse events 1% [0–3] • Lack of efficacy 11% [5–17] • Non-compliance 2% [0–5] • Other 2% [0–5] • Withdrew consent 1% [0–3] • Lost to follow up 1% [0–3] Time to discontinuation: • Before 6 months: 14% [7–21] • During 6–12 months: 18% [11–26] Still on treatment at the end of follow–up: 82% [74–89]	NA	Grade 3/4 adverse drug reactions: Lipase 7% [2–12] Amylase 34% [24–43] ALT 7% [2–12] Bilirubin 9% [4–15] Adverse drug reactions: 86% [79–93] Rash 11% [5–17]	NA	Diarrhea 11% [5–17] Nausea and vomiting 21% [13–29]	No death reported.	NA	HIV VL at 12 months (copies/mL): <50 46.5%, <400 66%
Rusconi et al. (25)	NA	NA	NA	NA	NA	NA	Median CD4 count at baseline (cells/mL): 364 [217–478] Median CD4 count at 42 months (cells/mL): 618 [534–684]	HIV VL <50 copies/mL at 42 months: 100%
Strehlau et al. (22)	All–causes: 16% [7–26] Related reasons: • Adverse events 9% [2–16] • Lack of efficacy 4% [0–8] • Non-compliance 2% [0–5] • Withdrew consent 2% [0–5] On treatment at end of follow–up: 84% [74–94]	NA	Grade 3/4 adverse drug reactions: Lipase 4% [0–9] Amylase 26% [15–38] ALT 11% [3–20] Bilirubin 9% [2–17] Adverse drug reactions: 93% [86–99]	NA	Diarrhea 36% [23–48] Nausea and vomiting 29% [17–40]	No death reported	Mean CD4 count at baseline (cells/mL): 1193 (sd:1004) Change CD4 count from baseline at 12 months (*n* = 29): +397 cells/mL (sd:363) or +7% in mean CD4%	HIV VL at 12 months (copies/mL): <50: 61%, <400: 74%
Bailey et al. (23)	All causes: 19% [15–23] Related reasons: • Adverse events 21% [12–23] • Lack of efficacy 20% [11–29] • Non-compliance 8% [2–15] • Patient wish 13% [5–20] • Simplified treatment 13% [5–20]	All causes: 11% [8–14] Related reasons: • Adverse events 10% [2–19] • Lack of efficacy 17% [6–27] • Non-compliance 10% [2–19] • Patient wish 8% [1–16] • Simplified treatment 8% [1–16] • Physician's decision 2% [0–6] • Unknown 31% [18–44] • Death 10% [2–19]	Grade 3/4 adverse drug reactions: 84% [80–89] Grade 3/4 hyperbilirubinemia: 49% [42–56]: (92/188, 6 with drug interruption). Grade 3/4 lipase: 2% [0–6] Grade 3/4 ALT: 2% [0–4]	Grade 3/4 adverse drug reactions: 82% [78–86] Grade 3/4 hyperbilirubinemia: 5% [1–15] (2/43) Grade 3/4 lipase: 0% Grade 3/4 ALT: 0.6%	NA	NA	NA	NA
	•Physician's decision 1% [0–4] • Unknown 20% [11–29] • Death 1% [0–4] • Pregnancy 1% [0–4]	
	Time to discontinuation (months): • <1: 7% [1–13] • 1– <6: 25% [15–36] • 6– <12: 17% [8–26] • ≥12: 51% [39–62]	Time to discontinuation (months): • <1: 13% [3–22] • 1– <6: 23% [11–35] • 6– <12: 21% [9–32] • ≥12: 42% [28–56]	
	Still on treatment at the end of follow–up: 81% [77–85]	Still on treatment at the end of follow–up: 89% [86–92]	

* *No comparator group was available for these outcomes*.

*ALT, Alanine Aminotransferase; ART, Antiretroviral drugs; ATV, Atazanavir; DRV, Darunavir; FAPV/r, Fosamprenavir/ritonavir; HIV, Human Immunodeficiency Virus; IQR, Interquartile Range; LPV/r, Lopinavir/ritonavir; NA, Not available; NS, Not significant; SD, Standard deviation; US, United States; VL, Viral Load*.

### Treatment Discontinuation

One single arm clinical trial by Strelhau et al. (22) reported treatment discontinuation for 16% (9/56) of patients, 9% (5/56) due to adverse events, 4% (2/56) to lack of viral suppression, 2% (1/56) to non-compliance and 2% (1/56) to withdrew consent. A non-comparative cohort study by Cotton et al. (24) reported treatment discontinuation for 18% (18/99) of patients: reasons for discontinuation included 1% (1/99) due to adverse events, 2% (2/99) to non-compliance and 11.1% (11/99) to lack of viral suppression. The only comparative cohort study by Bailey et al. (23) reported significantly higher all-cause-related treatment discontinuation in the atazanavir treatment group compared to the darunavir group, 19% (71/372) vs 11% (48/431) respectively (*p* = 0.002), with significant difference due to adverse events (*p* = 0.009) but not lack of efficacy (*p* = 0.10) or non-compliance (*p* = 0.58). The main adverse events reported among patients stopping atazanavir were renal, gastrointestinal, hematological or unspecified/other.

Thus, three studies (22–24) reported all-cause discontinuation rates, varying between 16% and 19%, yielding a homogeneous pooled incidence of 19% [15–22%] after 12 months of follow-up, (I^2^ 0%, *p* = 0.85). Overall, 81 to 84% were still on atazanavir at the end of follow-up.

### Grade 3 or 4 Adverse Events

Grade 3 or 4 adverse drug reactions were reported in four studies (22–24, 26) with rates varying from 10 to 93%. In the single comparative cohort study by Bailey et al. (23), 12 months after ART initiation, grade 3 or 4 events were frequently reported: 84% (226/268) of patients on atazanavir vs 82% (213/261) of those exposed to darunavir, with no significant difference. In two other studies with 12 months of follow-up after ART initiation, rates varied from 86% (85/99) (24) to 93% (52/56) (22). In a clinical trial with a shorter period of follow-up (6 months) by Piatt et al. (26), 10% (1/10) of the patients presented with grade 3 or 4 adverse events. No pooled incidence was presented due to significant heterogeneity between studies (I^2^ 95.6%, *p* = 0.00).

The grade 3 or 4 adverse events included plasma biological abnormalities. First, increased plasma lipase rates were observed in three studies with 12 months of follow-up after ART initiation, in 4% (2/53) (22), 2% (1/52) (23) and 7% (7/98) (24) of cases, yielding an estimated pooled incidence of 4% [1–7%] (I^2^ 23.7%, *p* = 0.27). Increased plasma alanine aminotransferase (ALT) rates were found in three studies in 2% (4/187) (23), 7% (7/98) (24) and 11% (6/53) (22) of patients. No cases of increased plasma ALT were reported for one clinical trial by Piatt et al. (26) with 6 months of follow-up. No pooled incidence was presented due to significant heterogeneity (I^2^ 70.5%, *p* = 0.03). Increased plasma amylase rates were reported in two studies (22, 24) for 26% (14/53) and 34% (33/98) of the cases, with a homogeneous pooled incidence of 31% [24–38%] (I^2^ 0%, *p* = 1.0). Last, increased hyperbilirubinaemia rates were found in four studies, varying from 9–10% (5/53, 1/10 and 9/98 respectively) (22, 24, 26) to 49% (92/188) (23) of patients, with significant heterogeneity (I^2^ 96.5%, *p* < 10^−4^).

For the single comparative study by Bailey et al. (23) reporting data for atazanavir compared to darunavir, there were few adverse events and proportions were comparable for the two drugs, except for transient hyperbilirubinaemia found in 49% (92/188) of the patients exposed to atazanavir vs. 5% (2/43) of those receiving darunavir. Among patients on atazanavir, hyperbilirubinemia declined significantly over time (66/100 person-year before 12 months to 32/100 after 24 months, *p* < 10^−4^). Six over the 92 cases lead to treatment discontinuation. There was no significant difference in grade 3 or 4 adverse event rates (*p* = 0.405), abnormal lipase (*p* = 0.634) or ALT rates (*p* = 0.207) between groups.

### Others Adverse Events

Other adverse events were reported in four studies (22–24, 26). In two studies with 12 months of follow-up after antiretroviral treatment initiation, adverse events were frequently reported and ranged from 86% (85/99) to 93% (52/56) of patients (22, 24).

Diarrhea was reported in two studies (22, 24), with rates varying between 11% (11/99) and 36% (20/56) at 12 months of antiretroviral treatment, yielding a homogeneous pooled incidence of 16% [10–21%] (I^2^ 0%, *p* = 1.0). These studies also documented nausea and vomiting, with rates between 21% (21/99) and 29% (16/56), and a homogeneous pooled incidence of 24% [17–30%] (I^2^ 0%). Only one cohort study by Cotton et al. (24) documented rash in 11% (9/99) of patients.

### Death

No death occurred in the two studies reporting this event (22, 24).

### Effectiveness of Atazanavir Use in Children and Adolescents Living With HIV

Two non-comparative studies out of the five reported increased CD4 cell counts after antiretroviral treatment initiation, compared to baseline. A retrospective cohort by Rusconi et al. (25) reported an increase in mean CD4 absolute number per μl from 364 [217–478] to 618 [534–684] at 42 months of follow-up (*p* < 0.05). In a phase 3b clinical trial by Strelhau et al. (22), the median change from baseline in CD4 cell count was +363 cells/mm3 or +7.5% in CD4 percent after 24 months of follow-up. Another, an open-label clinical trial by Piatt et al. (26) found stable mean CD4 cell count over time (mean CD4 cell count at baseline 1233 [SD 473.8]; to 1120 [SD 413.2] at 6 months).

Four studies out of five assessed viral response at different time points, none of them using a comparator. One open-label clinical trial by Piatt et al. (26) reported no HIV viral load failure (≥200 copies/mL) at 6 months among all 10 patients. HIV viral load was undetectable (<50 copies/mL) at 12 months in two studies for 47% (46/99) and 61% (33/45) of patients (22, 24), yielding a pooled incidence of 52% (44–60%) (I^2^ 0%). Also, in these studies, HIV viral load was <400 copies/mL in 66% (65/99) and 74% (40/45) of patients, with a pooled incidence of 69% (62–77%) (I^2^ 0%). The small retrospective cohort by Rusconi et al. (25) reported an undetectable viral load <50 copies/mL among all seven patients treated over the 42 months of follow-up.

### Quality Assessment

Quality assessment is summarized in Table 3.

**Table 3:** Assessment of risk of bias for included studies for the atazanavir review.

**Study**	**Study design**	**Randomization process**	**Deviations from the intended interventions**	**Missing outcome data**	**Outcome assessment**	**Selection of the reported result**	**Overall risk of bias summary**
Cotton et al. (24)	Prospective, international, non-comparative, single arm phase 3b trial	High risk	No comparator	Not reported: High risk	Low risk	Low risk	High risk
Piatt et al. (26)	Prospective, non-comparative, open–label clinical trial	High risk	No comparator	Low risk	Low risk	Low risk	High risk
**Study**	**Study design**	**Selection of study population**	**Exposure assessment**	**Outcome assessment**	**Prognostic factors assessment**	**Follow–up of cohort adequate**	**Overall risk of bias summary**
Rusconi et al. (25)	Retrospective non-comparative cohort	No comparator	Low risk	Low risk	Unclear	Unclear	High risk
Strehlau et al. (22)	Prospective cohort based on a phase 3b clinical trial	No comparator	Low risk	Low risk	Unclear	Low risk	High risk
Bailey et al. (23)	Retrospective comparative cohort	Low risk	Low risk	Low risk	Low risk	Unclear	Moderate risk because not randomised

The two clinical trials (24, 26) were classified as having high risk of bias, because no randomization process was applied, no comparator was available, and one phase 3b clinical trial by Strelhau et al. (22) did not report information about missing data. Moreover, no sample size calculation was reported.

Among the three cohort studies, two non-comparative (22, 25) were categorized as high risk of bias, due to unclear assessment of prognostic factors, no sample size calculation and small study population (<100). The single comparative cohort study by Bailey et al. (23) was classified as moderate risk because no randomization process was applied to determine atazanavir or darunavir arm.

## Discussion

Limited safety and effectiveness data were available for children and adolescents living with HIV receiving atazanavir-based treatment. Five studies have been included in this systematic review, with a single cohort study comparing atazanavir to darunavir for safety only (23).

First, in terms of safety, we estimated the pooled incidence rates for three types of safety data (treatment discontinuation, grade 3 or 4 adverse events including plasma biological abnormalities, and other adverse events). Most of the patients were still on treatment at the end of follow-up (over 80%). Overall, few patients discontinued treatment due to toxicity. However, the single comparative cohort study found a significantly higher all-cause treatment discontinuation rate in the atazanavir treatment arm compared to the darunavir arm, explained by adverse events but not by lack of efficacy or non-compliance (23). Moreover, in this study, the most frequently reported adverse event was grade 3 or 4 hyperbilirubinemia, occurring in half of the patients but declining over time (23). Several studies have reported increased rates of hyperbilirubinemia after atazanavir exposure in adults living with HIV (27–29). Since atazanavir inhibits the uridine diphosphate glucuronosyltransferase (UGT), responsible for bilirubin conjugation, patients living with HIV with the UGT1A1^*^28 allele present a high risk of hyperbilirubinemia (15, 30). In this case, WHO recommends switching to another antiretroviral drug if adherence is compromised (30). However, this higher risk of grade 3 or 4 hyperbilirubinemia was reported in only one study, while three others studies reported lower rates (around 9–10%). It is possible that differences in the prevalence of the polymorphism UGT1A1^*^28 might explain these different findings (22, 24, 26).

In addition, the most frequently reported safety outcome was grade 3 or 4 adverse events, but the results were heterogeneous with rates varying from 84% in the comparative cohort study (23) to 10% in an open-label clinical trial (26). This low rate may be explained by the shorter follow-up period (only 6 months vs 12 months of follow-up) and the smaller sample size (only 10 patients), leading to a lower probability of observing adverse events. Grade 3 or 4 adverse events consisted mainly of plasma biological abnormalities, and were uncommon, with the exception of high rates of grade 3 or 4 hyperbilirubinemia as previously mentioned. Other adverse events were reported in a majority of patients in two studies conducted in children (22, 24), but these were not classified as grade 3 or 4 events. The main other adverse outcomes were diarrhea, and nausea and vomiting, with consistent pooled incidence of 16 and 24%, respectively. Those outcomes are the most commonly adverse drug reactions observed with antiretroviral therapy. Lastly, no death occurred in the two studies reporting this event (22, 24).

Among the four studies of the five reporting effectiveness data, none used a comparator. Undetectable HIV viral load (<400 or 50 copies/mL), and increased or stable CD4 cell counts after atazanavir exposure were reported. These results were consistent with those found in adults living with HIV (31–34). Nonetheless, the lengths of follow-up on atazanavir were heterogeneous between studies, varying from 6 to 42 months. Consequently, CD4 cell counts were stable in the 6-months clinical trial (26), while increased CD4 cell counts were reported beyond 24 and 42 months of follow-up (22, 25). Moreover, in two among the four studies reporting effectiveness data, the sample size was very small [seven and 10 patients respectively (25, 26)]. As a result, this systematic review described good virological responses (CD4 cell count and HIV viral load), that need to be further documented.

Our study has some limitations. Few studies were included, among which sample sizes were small (<100 patients, with two studies that enrolled around 10 patients) except for one comparative cohort (23). Follow-up time after ART initiation and characteristics of the study population varied greatly between studies. Only one study included a high proportion of treatment naïve patients (61%, 34/56) (22). Furthermore, two studies included patients exposed to didanosine and stavudine, two antiretroviral drugs that are no longer recommended for treatment of children and adolescents due to adverse events such as lipoatrophy, lactic acidosis, peripherical neuropathy and pancreatitis (35–37). As atazanavir has been used since 2004, several children and adolescents were exposed to antiretroviral regimens based on this protease inhibitor drug and two NRTIs, mainly zidovudine, lamivudine, stavudine, didanosine or tenofovir. Moreover, one cohort study (25) has evaluated the effectiveness of a dual therapy, including fosamprenavir and atazanavir, that could influence the increase of side effects. However, no safety data were described, which is limiting our interpretation, as the rates of adverse events could differed according to protease inhibitor drugs used. Last, four of the five studies presented some concerns of bias due to lack of randomization, lack of information on missing data, lack of assessment of prognostic factors or small sample size without validated calculation. Despite those limitations, this systematic review is, to our knowledge, the first to document the safety and effectiveness of atazanavir in children and adolescents living with HIV. We searched published as well as unpublished studies, regardless of their results or their study place, to increase the representativeness and to limit publication bias.

## Conclusion

Limited safety and effectiveness data were available for children and adolescents living with HIV exposed to atazanavir. In terms of safety, few grade 3 or 4 adverse events were reported, except for high rates of transient hyperbilirubinemia. Other adverse events (diarrhea, nausea) were common, but over 80% of patients were still on treatment at the end of follow-up. Available descriptive effectiveness results showed undetectable HIV viral load (<400 or 50 copies/mL), and increased or stable CD4 cell counts after atazanavir exposure. Further long-term and comparative studies are needed on atazanavir use in children and adolescents living with HIV. Despite limited evidence, the use of atazanavir/ritonavir in children and adolescents living with HIV remains a good option for a preferred second-line antiretroviral regimen, as an alternative to lopivanir/ritonavir, with good virological responses. Our results are in line with the revised WHO guidelines (30).

## Data Availability Statement

The original contributions presented in the study are included in the article/Supplementary Material, further inquiries can be directed to the corresponding author.

## Ethics Statement

Ethical review and approval was not required for the study on human participants in accordance with the local legislation and institutional requirements.

## Author Contributions

LSL, MD, and VL contributed to the search strategy and selection of relevant studies. LSL and MD independently screened the abstracts identified. LSL extracted the data of each selected study and undertook the statistical analysis. LSL, MD, JJ, and VL performed methodological quality analysis. LSL wrote the first draft of the manuscript that all co-authors reviewed and edited. All authors contributed to the development of the study protocol. All authors contributed to the article and approved the submitted version.

## Funding

WHO the interpretation of results expressed in this article arose from the authors and do not reflect the views of the funder. All authors had access to the data of the study.

## Conflict of Interest

The authors declare that the research was conducted in the absence of any commercial or financial relationships that could be construed as a potential conflict of interest.

## Publisher's Note

All claims expressed in this article are solely those of the authors and do not necessarily represent those of their affiliated organizations, or those of the publisher, the editors and the reviewers. Any product that may be evaluated in this article, or claim that may be made by its manufacturer, is not guaranteed or endorsed by the publisher.

## Abbreviations

95%CI: 95% Confidence Intervals
ALT: alanine aminotransferase
CLARITY: Clinical Advances through Research and Information Translation
CRD: Centre for Reviews and Dissemination
HIV: Human Immunodeficiency Virus
PRISMA: Preferred Reporting Items for Systematic Review and Meta-Analysis
WHO: World Health Organization.
