# Supplementary material for: Effectiveness and Safety of Atazanavir Use for the Treatment of Children and Adolescents Living With HIV: A Systematic Review
Source: Front Pediatr. 2022 May 23;10:913105. doi: 10.3389/fped.2022.913105 (PMC9168429; doi:10.3389/fped.2022.913105)
Supplement: Supplementary file 1 [file Table_1.DOCX]

This additional file presented the search strategies for published and unpublished records.

**Search strategies for published studies**

Search strategies for Web of Science, the Cochrane Library (CENTRAL) and Embase are presented in Tables 1, 2 and 3 respectively.

**Table 1**: Search strategy for Web of Science between 2009 to 2020 (2009/01/01-2020/10/01)

| **#** | **Searches** | **Results** |
| --- | --- | --- |
| 1 | HIV | 266797 |
| 2 | HIV Infections* | 135638 |
| 3 | (HIV or HIV1 or HIV-1 or HIV2 or HIV-2 or human immun?deficiency virus* or human immun? deficiency virus*) | 284775 |
| 4 | (AIDS or acquired immun? deficiency syndrome* or acquired immun?deficiency syndrome*) | 651292 |
| 5 | #1 or #2 or #3 or #4 | 860187 |
| 6 | Atazanavir/ritonavir | 501 |
| 7 | atazanavir  sulfate  OR  atazanavir  OR  atazanavir/ritonavir  OR  ATV  OR  ATV/r | 5340 |
| 8 | Atanazavir/ritonavir or CGP73547 or CGP 73547 or BMS23263205 or BMS 232632 05 or CGP 75176 or CGP75176 or Reyataz or CGP75355 or CGP 75355 or BMS232632 or BMS 232632 or CGP 75136 or CGP75136 | 21 |
| 9 | #6 or #7 or #8 | 5343 |
| 10 | (adolescen* or babies or baby or boy? or boyfriend or boyhood or girlfriend or girlhood or child* or girl? or infan* or juvenil* or kid? or minors or minors* or neonat* or neo-nat* or newborn* or new-born* or paediatric* or peadiatric* or pediatric* or perinat* or preschool* or puber* or pubescen* or school* or teen* or toddler? or underage? or under-age? or youth* or young) | 4301049 |
| 11 | (pediatric* or paediatric* or infan* or child* or adolescen* or young) | 3435321 |
| 12 | #10 or #11 | 4301049 |
| 13 | #5 and #9 and #12 | 453 |
| 14 | (Case report* or case stud*) | 2616403 |
| 15 | #13 not #14 | 399 |
| 16 | #15 has an abstract | 375 |
| 17 | #16 remove duplicates | 373 |

**Table 2:** Search strategy for Cochrane Library (CENTRAL) run on 25 September 2020 (2009/01/01 to 2020/09/25)

| **ID** | **Search** | **Hits** |
| --- | --- | --- |
| #1 | MeSH descriptor: [HIV] explode all trees | 3034 |
| #2 | MeSH descriptor: [HIV Infections] explode all trees | 12403 |
| #3 | ((HIV or HIV1 or HIV-1 or HIV2 or HIV-2 or human immun?deficiency virus* or human immun? deficiency virus*)) | 43516 |
| #4 | (AIDS or acquired immun? deficiency syndrome* or acquired immun?deficiency syndrome*) | 17178 |
| #5 | (1-#4) | 48643 |
| #6 | MeSH descriptor: [Atazanavir sulfate] explode all trees | 287 |
| #7 | atazanavir  sulfate  OR  atazanavir  OR  atazanavir plus ritonavir  OR  ATV  OR  ATV plus r | 1146 |
| #8 | atazanavir sulfate or CGP73547 or CGP 73547 or BMS23263205 or BMS 232632 05 or CGP 75176 or CGP75176 or Reyataz or CGP75355 or CGP 75355 or BMS232632 or BMS 232632 or CGP 75136 or CGP75136 | 350 |
| #9 | (#5-#8) | 1147 |
| #10 | pediatric* or paediatric* or infan* or child* or adolescen* or young | 350194 |
| #11 | (adolescen* or babies or baby or boy? or boyfriend or boyhood or girlfriend or girlhood or child* or girl? or infan* or juvenil* or kid? or minors or minors* or neonat* or neo-nat* or newborn* or new-born* or paediatric* or peadiatric* or pediatric* or perinat* or preschool* or puber* or pubescen* or school* or teen* or toddler? or underage? or under-age? or youth* or young) | 438773 |
| #12 | (#6-#23) | 438773 |
| #13 | #5 AND #9 AND #12 | 186 |
| #14 | #13 with Publication Year from 2009 to 2020, in Trials | 152 |
| #15 | #13 in Cochrane Reviews | 7 |
| #16 | #14 or #15 | 159 |
| #17 | #16 remove duplicates | 157 |

**Table 3:** Search strategy for EMBASE between 2009 to 2020, run on 6 October 2020

| **#** | **Searches** | **Results** |
| --- | --- | --- |
| 1 | exp HIV/ | 196838 |
| 2 | exp HIV Infections/ | 379074 |
| 3 | (HIV or HIV1 or HIV-1 or HIV2 or HIV-2 or human immun?deficiency virus* or human immun? deficiency virus*).mp. | 505845 |
| 4 | (AIDS or acquired immun? deficiency syndrome* or acquired immun?deficiency syndrome*).mp. | 244083 |
| 5 | or/1-4 | 606905 |
| 6 | Atanazavir plus ritonavir or CGP73547 or CGP 73547 or BMS23263205 or BMS 232632 05 or CGP 75176 or CGP75176 or Reyataz or CGP75355 or CGP 75355 or BMS232632 or BMS 232632 or CGP 75136 or CGP75136.mp. | 719 |
| 7 | (atazanavir sulfate or atazanavir or atazanavir plus ritonavir or ATV or ATV plus r).mp. | 10045 |
| 8 | Or/6-7 | 10048 |
| 9 | (adolescen* or babies or baby or boy? or boyfriend or boyhood or girlfriend or girlhood or child* or girl? or infan* or juvenil* or kid? or minors or minors* or neonat* or neo-nat* or newborn* or new-born* or paediatric* or peadiatric* or pediatric* or perinat* or preschool* or puber* or pubescen* or school* or teen* or toddler? or underage? or under-age? or youth* or young).ti,ab,kw. | 3616301 |
| 10 | (pediatric* or paediatric* or infan* or child* or adolescen* or young).jn,jw. | 803332 |
| 11 | or/9-10 | 3796467 |
| 12 | 5 and 8 and 11 | 748 |
| 13 | (Case report* or case stud*).mp. | 2733149 |
| 14 | 12 not 13 | 687 |
| 15 | limit 14 to yr=”2009 – Current” | 558 |
| 16 | Limit 15 to abstracts | 530 |
| 17 | Remove duplicates from 16 | 521 |

**Search strategies for the grey literature**

Search strategies for conference material and clinical guidelines were performed on clinical trial registries updated in the past two years (ClinicalTrials.gov; WHO International Clinical Trials Registry Platform; EudraCT), on the references from the most recent international guidelines on HIV treatment (WHO antiretroviral guidelines (6), US National Institutes of Health Guidelines for the Use of Antiretroviral Agents in Pediatric HIV Infection 2020 (15), Penta 2015, 2016 and 2019 guidelines (16–18)) and on conference abstract books (International AIDS Society Conference 2019 and 2020, Conference on Retroviruses and Opportunistic Infections 2019 and 2020, International Workshop on HIV Pediatrics 2018 and 2019).

Conference abstract books will be searched for each of the relevant drug names and the most common abbreviation (Table 2) in the following conferences from the previous two years:

- - International AIDS Society Conference 2019 and 2020 (2019 abstracts available from: <http://programme.ias2019.org/Abstract>; 2020 abstracts available from https://www.aids2020.org/wp-content/uploads/2020/09/AIDS2020_Abstracts.pdf)
  - Conference on Retroviruses and Opportunistic Infections (CROI; 2019 [<https://www.croiconference.org/croi-2019/>] and 2020 [<https://www.croiconference.org/croi-2020/>])
  - International Workshop on HIV Pediatrics (2018 and 2019 abstract books available from <http://www.infectiousdiseasesonline.com/abstract-book/>)
  - International Conference on AIDS and STIs in Africa 2019 (Abstract book available from <https://saafrica.org/new/wp-content/uploads/2020/02/ICASA-2019-Abstract-Book-online-version.pdf>)

Clinicaltrial.gov search strategy

In the advanced search settings (<https://clinicaltrials.gov/ct2/search/advanced>), the following terms will be used:

**Condition or disease:** HIV

**Study type:** All studies

**Study results:** All studies

**Age group:** Child (birth-17)

**Intervention/treatment:** Atazanavir OR ATV

**Last Update Posted:** From 01/01/2018; To 09/21/2020

All other fields will be left at the default settings. Test search retrieved 72 studies (run 21 September 2020).

WHO International Clinical Trials Registry search strategy

In the advanced search settings (<https://apps.who.int/trialsearch/AdvSearch.aspx>), the following terms will be used:

**In the Condition:** HIV

**In the Intervention:** Atazanavir OR ATV

Select tick box for “Search for clinical trials in children”. All other settings used will be default. The search results will be exported into Excel and only trials which were “Last refreshed on” 2018 to Present will be retained. Trials with NCT registration (i.e. from clinicaltrials.gov) will be removed as the clinicaltrials.gov website will be more up to date.

EudraCT search strategy

In the advanced settings <https://www.clinicaltrialsregister.eu/ctr-search/search>, the following terms will be used:

**Search terms:** Atazanavir OR ATV

**Age range:** Adolescent and children and infant and toddler and newborn and preterm new born infants and under 18

Click on “Advanced Search: Search tools” to define age range above. Test search retrieved 17 trials on 21 September 2020.
